# Defining Multi-Trait Breeding Objectives and Selection Indexes to Develop More Efficient Breeding Programs for Superfine Wool Sheep

**DOI:** 10.3390/ani15131873

**Published:** 2025-06-24

**Authors:** Tingting Guo, Wenhui Li, Chao Yuan, Xijun Wang, Jianbin Liu, Bin Liang

**Affiliations:** 1 Key Laboratory of Animal Genetics and Breeding on the Tibetan Plateau, Ministry of Agriculture and Rural Affairs, Lanzhou Institute of Husbandry and Pharmaceutical Sciences, Chinese Academy of Agricultural Sciences, Lanzhou 730050, China; yuanchao@caas.cn (C.Y.); liujianbin@caas.cn (J.L.); 2 Sheep Breeding Engineering Technology Research Center of Chinese Academy of Agricultural Sciences, Lanzhou 730050, China; 3 Gansu Provincial Sheep Breeding Technology Extension Station, Huang Cheng Town, Sunan 734031, China; whleee2004@163.com (W.L.); wxj18993661572@163.com (X.W.); liangbin2025@126.com (B.L.)

**Keywords:** Alpine Merino sheep, superfine strain, breeding objective, multi-trait selection index, genetic gain

## Abstract

This study aimed to develop an optimized breeding program for the Alpine Merino sheep superfine strain by defining breeding objectives and establishing a multi-trait selection index. Using phenotypic and genetic (co)variance components of wool and growth traits, we identified six key breeding objectives: average fiber diameter (AFD), clean fleece weight (CFW), staple strength (SS), staple length (YSL), yearling weight (YWT), and weaning weight (WWT), with economic weights of 48, 48.5, 2, 12, 2.85, and 25, respectively. Through eco-bio modeling comparing three index sets with different trait combinations and information sources, we determined the optimal index comprising the AFD, CFW, fiber diameter coefficient of variation (FDcv), YSL, YWT, and WWT using data from individual phenotypes and 10 half-sibs. Recommended ram and ewe utilization periods were 3 and 5 years, respectively. Projected 10-year genetic gains showed improvements of −2 μm (AFD), 0.57 kg (CFW), 20 N/ktex (SS), 0.6 cm (YSL), 4.73 kg (YWT), and 0.38 kg (WWT). The developed program enhances both superfine wool characteristics and overall wool quality while improving the selection accuracy. Additionally, we propose alternative indices for varying measurement contexts and discuss strategies to accelerate genetic gains, including optimizing ewe reproduction rates and ram selection precision, and incorporating progeny information.

## Simple Summary

As the direction of the selection of fine wool sheep is shifting toward ultrafine, in order to better achieve selection goals, a breeding program for ultrafine lines of Alpine Merino sheep was developed by identifying six key breeding objectives, average fiber diameter (AFD), net fleece weight (CFW), short staple strength (SS), short staple length (YSL), yearling weight (YWT), and weaning weight (WWT), in order to establish a multi-trait selection index for ultrafine lines of Alpine Merino sheep. It improves both the characteristics and the overall wool quality of ultrafine wool, as well as the accuracy of the selection. In addition, we present alternative indices for different measurement environments and discuss strategies to accelerate genetic gain, including optimizing ewe fertility and ram selection accuracy, and incorporating progeny information.

## 1. Introduction

Fine wool sheep breeding aims to enhance product quality and quantity while maintaining environmental adaptability and improving enterprise profitability. In production systems, this involves multiple economically important traits that require precise breeding objectives (aggregate genotypes) and multi-trait selection indices to maximize genetic gains. Australia’s merino breeding program, a global leader, has implemented genetic evaluation systems for over 50 years. WOOLPLAN, which was released in 1987 [1], introduced aggregate genotypes and a multi-trait selection index based on economically important traits. Initially WOOLPLAN was able to obtain breeding values of five traits and four selection indexes [2], and progressively incorporated additional traits, including fly strike traits [3], early skin follicle traits [4], and meat production traits [5,6,7] into the sheep genetic evaluation system. As the genetic evaluation system developed, new selection indexes were established, catering to different Merino breeding directions. The current MERINOSELECTION system integrates 87 traits and 29 selection indices [8,9], demonstrating how continuous genetic evaluation optimization drives Merino sheep improvement and industry sustainability. In China, similar breeding objective research [10,11,12] has helped align domestic practices with international standards. Our research team bred Gansu Alpine fine wool sheep in the 1980s through crossbreeding and intensive selection [13]. The subsequent introduction of Australian superfine Merinos led to the 2015 establishment of Alpine Merino sheep as an upgraded breed [14]. Breeding priorities have evolved significantly, shifting from an early emphasis on body weight, staple length, and fleece weight [15] to the current focus on fiber diameter, clean fleece weight, and superfine wool characteristics [13,16,17]. Current evaluations show that 51% of ewes produce wool measuring 14~20 μm (including 7% below 16 μm) (*n* = 1597), with concurrent improvements in other traits. The selection of a new ultra-fine wool sheep population from the core flock of Alpine Merino sheep has undoubtedly become an invaluable genetic resource and brings greater economic value [18]. However, existing breeding programs lack comprehensive bioeconomic models to quantify trait contributions or optimize multi-trait selection indices. This study addresses these gaps by developing integrated breeding objectives and efficient selection indices specifically for the Alpine Merino ultra-fine wool sheep population, with the potential to significantly improve selection accuracy and genetic gain efficiency.

## 2. Materials and Methods

### 2.1. Breeding and Production Management System

This study was conducted at the Gansu Provincial Sheep Breeding Technology Extension Station, which maintains a nucleus population of 120,000 Alpine Merino sheep distributed across multiple household-managed flocks. The breeding program consists of one breeding ram flock maintained at one hundred twenty heads annually and fifteen reproduction ewe flocks totaling five hundred fifty heads, including five nucleus ewe flocks (three ultra-fine flocks and two fine flocks). The flocks exhibit an average reproduction rate of 85%, with the highest-performing households achieving 105%. Current culling ages are set at 6 years for rams and 7 years for ewes. The station implements a standardized breeding protocol featuring annual artificial insemination from late November to early December, resulting in lambing between mid-April and May. This process employs a hierarchical assortative mating system where parental selection is carefully matched to specific strain requirements. The comprehensive evaluation system begins with early assessments, including pedigree recording at birth for all lambs from nucleus flocks, followed by weaning evaluations in August that focus on body weight and weaning grade. From these, approximately 2250 replacement ewe lambs (150 per rearing mob) and 100 ram lambs are selected annually. Yearling assessments conducted each June incorporate detailed wool quality evaluations, with mid-side wool samples analyzed by authorized fiber testing institutes for the average fiber diameter (AFD), fiber diameter coefficient of variation (FDcv), and wool yield (YLD). Following shearing in early July, individual greasy fleece weights (GFWs) are recorded, and clean fleece weights (CFWs) are calculated as the product of the GFW and yield. These measurements form the basis for the final strain classification and selection decisions.

Genetic analyses using ASReml’s BLUP linear mixed animal model, drawing on two decades of data encompassing approximately 50,000 records, have demonstrated significant genetic progress. Notable improvements include a reduction in the AFD from 19.95 μm to 17.92 μm, an increase in body weight from 38.17 kg to 41.07 kg, enhanced CFW from 2.17 kg to 2.32 kg, and improved yield from 46.88% to 60.71%. All animal husbandry and measurement procedures were conducted with the approval of the Institutional Animal Care and Use Committee at the Lanzhou Institute of Husbandry and Pharmaceutical Sciences, with every effort made to minimize animal discomfort throughout the study.

### 2.2. Estimation of Genetic Parameters

Data from 49,474 Alpine Merino sheep raised at the Gansu Sheep Breeding Technology Extension Station were analyzed in a previous study [19]. The dataset included birth weight records from animals born between 2000 and 2019, representing the progeny of 526 sires and 22,531 dams. The analysis employed restricted maximum likelihood (REML) methods implemented in ASReml [20] to fit a linear mixed animal model. For each trait, the model incorporated fixed effects (where significant) for birth type, dam age, sex, management group, record age, and birth year, along with various random effects. Notably, the interaction between sire genotype and dam strain flock was treated as a genetic group effect and included as a random component. Model selection was performed using log-likelihood ratio tests, comparing seven alternative model configurations with different combinations of random effects. Detailed methodological descriptions are available in the original publications [19,21]. A subsequent study [21] extended this work by estimating the heritability of the wool staple strength and its phenotypic and genetic correlations with other traits using additional data from 2017 to 2018 that included wool staple strength measurements. Table 1 summarizes the key parameters estimated in these two studies, which serve as the foundation for the current investigation.

### 2.3. Identification of Breeding Objectives and Economic Weights

The breeding strategy for the superfine strain of Alpine Merino Sheep aims to enhance wool quality—particularly the fiber diameter—while maintaining or increasing body weight and clean fleece weight, without compromising the breed’s adaptability to high-altitude, cold environments. The selection criteria for breeding objectives were determined based on this overarching goal, as well as historical genetic progress in economically important traits. A simplified production model was employed to estimate the economic weights of breeding objective traits, simulating a single reproductive ewe flock over one production cycle. The economic weight for each trait was derived from its marginal benefit, defined as the net income generated per unit improvement in the trait (i.e., total revenue minus associated costs) [10,11]. For wool production traits (e.g., fleece weight), the economic weight reflects the net income change resulting from a one-unit increase in yield. For wool quality traits (e.g., fiber diameter), it corresponds to the price premium associated with a unit improvement in the trait (Table 2). The economic weights were adjusted from their marginal benefit values to accommodate practical breeding constraints, incorporating both the desired gain approach [3,22,23] for realistic selection progress and balanced emphasis across breeding objectives. The breeding objective also defined as an aggregate genotype as follows:

(1) $$Aggregate\;genotype\left(H\right)=v_{1}g_{1}+v_{2}g_{2}+\cdots +v_{n}g_{n}$$

where v refers to economic weights of the objective traits 1 to n, and g refers to the deviation of breeding value of the relevant breeding objective trait. A parameter that represents the variation in the aggregate genotype (H) is $\sigma_{H}=\sqrt{v^{’}Cv}$ , where v refers to vector of economic weights of breeding objective traits, and C is the matrix of genetic variance and covariance of the breeding objective traits.

### 2.4. Index Construction and Evaluation

Three index trait groups (A, B, and C) were initially designed (Table 3), along with seven scenarios of information sources (Table 4), to determine the most suitable index selection program. This was achieved by comparing the index standard deviation (ϭI), selection accuracy and selection response across different index traits and information source scenarios. Group C comprised breeding objective traits: yearling body weight (YWT), weaning weight (WWT), yearling wool staple length (YSL), average fiber diameter (AFD), clean fleece weight (CFW), and wool staple strength (WSS). Group A included the same traits as Group C, except that staple strength (WSS) was replaced by the coefficient of variation of AFD (CVAFD). This tested the hypothesis that Group A could substitute Group C. Group B contained only assessment-stage traits—YWT, WWT, YSL, wool fineness count (VFC), and greasy fleece weight (GFW)—to evaluate whether conventional assessment traits could replace lab-measured wool traits, thereby reducing testing costs.

Index traits are the traits that the selection directly works on, and the multi-trait index is expressed as follows:

(2) $$Index\left(I\right)=b_{1}x_{1}+b_{2}x_{2}+\cdots +b_{m}x_{m}$$

The selection index (I) is calculated as follows:

(3) $$I=\sum b_{i}X_{i}$$

where

*Xi* represents the phenotypic performance of an animal (or the mean phenotypic performance of its relatives) for trait i and *bi* is the corresponding weighting factor.

The weight vector (b) is derived as follows:

(4) $$b=P^{-1}G_{v}$$

where

P is the phenotypic (co)variance matrix of the index traits; G is the genetic (co)variance matrix, with rows corresponding to index traits and columns to breeding objective traits; and v is the vector of economic weights for the objective traits.

These weights (b) account for the relative importance of each breeding goal trait, ensuring that ranking animals by the index optimally reflects their genetic merit. When the index incorporates both an animal’s own performance and its relatives’ performance, separate weights are calculated for each component. These computations were performed using the program developed by Julius van der Werf [22]. To compare the selection efficiency of different indices (varying in trait composition and information sources), two key metrics were used: the index standard deviation (σₐ)

(5) $$\sigma_{I}=\sqrt{b^{\prime }Pb}$$

and selection accuracy (*R_IH_*)

(6) $$R_{IH}=\frac{\sigma_{I}}{\sigma_{H}}$$

where σ_H_ is the standard deviation of the aggregate breeding value (H).

### 2.5. Flock Age Structure, Selection Intensity, and Impact of the Reproduction Rate on Genetic Gains

The flock age structure of the Alpine Merino Sheep nucleus population was determined based on the culling age (5–6 years for rams and 6–7 years for ewes with a minimum breeding age of 2 years) and a 4% annual mortality rate for both sexes. Breeding rams were selected from male lambs born in five nucleus ewe flocks (85% average reproduction rate), with replacement ewes also chosen from these flocks. Annual flock maintenance required retaining animals equivalent to previous year’s deaths plus culled animals. To optimize selection strategies, genetic gains were compared across different utilization periods. Furthermore, comparing annual genetic gains under index selection at 85% versus 95% reproduction rates revealed that higher reproduction increased selection candidates, enabling lower retention rates and a higher selection intensity while maintaining replacement needs, thus demonstrating how reproductive efficiency directly affects genetic improvement rates. This integrated analysis considered both demographic factors and reproductive performance in breeding program optimization.

### 2.6. Prediction of the Selection Response and Annual Genetic Gains

For a specific selection index, when the selection intensity is 1 in single selection round, the selection response for the ith trait (R) is given by the following:

(7) $$R=\frac{b\prime G_{i}}{\sigma_{i}}$$

(8) $$\mathrm{Annual}\;\mathrm{genetic}\;\mathrm{gain}\;\mathrm{of}\;\mathrm{the}\;\mathrm{ith}\;\mathrm{trait}:\;∆G_{i}=\frac{\frac{i_{m}{b^{\prime }}_{m}G_{m,i}}{\sigma_{I_{m}}}+\frac{i_{f}{b^{\prime }}_{f}G_{f,i}}{\sigma_{I_{f}}}}{L_{m}+L_{f}}$$

(9) $$\mathrm{Annual}\;\mathrm{genetic}\;\mathrm{gain}\;\mathrm{of}\;\mathrm{aggregate}\;\mathrm{genotype}:\;∆H=\frac{i_{m}\sigma_{I_{m}}+i_{f}\sigma_{I_{f}}}{L_{m}+L_{f}}$$

The selection response (R) is determined by the index weights (b) and the genetic architecture of the traits. Specifically, b represents the vector of weighting coefficients for index traits and G denotes the genetic variance-covariance matrix, with rows corresponding to index traits and columns representing all traits (both selection index traits and breeding objective traits).

Within this matrix structure:

G_i_ refers to the genetic covariance vector for the ith objective trait.
Subscripts m and f differentiate between male and female selection pathways.

For instance, G_m-i_ indicates the ith column of the genetic covariance matrix specific to male selection.

### 2.7. Selection Emphasis and Parental Contributions to Genetic Improvement

The relative importance of each objective trait in the breeding program was quantified as follows:

(10) $$Selection\;emphasis(\%)=\frac{\left[\Delta G_{i}\times v_{i}\times 100\right]}{∆H}$$

where

ΔG*_i_* is the annual genetic gain for the *i*th objective trait (from Equation (8));
v_i_ is the economic weight for the trait; and
Δ*H* is the annual genetic gain of the aggregate genotype (from Equation (8)).

Parental contributions to the aggregate genetic gain were calculated as follows:

(11) $$Sire\;contribution\;(\%)=\frac{\frac{i_{m}\sigma_{I_{m}}}{L_{m}+L_{f}}\times 100}{∆H}$$

(12) $$Dam\;contribution\;(\%)=\frac{\frac{i_{f}\sigma_{I_{f}}}{L_{m}+L_{f}}\times 100}{∆H}$$

where

$i_{m}$ and $i_{f}$ are selection intensities for males and females;
$\sigma_{I_{m}}$ and $\sigma_{I_{f}}$ are standard deviations of selection indices; and
$L_{m}$ and $L_{f}$ are generation intervals for each sex.

## 3. Results

### 3.1. Breeding Objective Traits and Corresponding Weights

The study established six economically significant traits as breeding objectives (Table 5): average fiber diameter (AFD), clean fleece weight (CFW), yearling body weight (YWT), weaning weight (WWT), wool staple strength (SS), and wool staple length (YSL). Economic weights for these traits were determined through a marginal benefit analysis using a production model (Table 5). The economic weights were determined as follows: YWT (2.85), WWT (15.1), and CFW (48.5) were calculated based on their direct production value. For wool quality traits, YSL and SS were both assigned a weight of 12, reflecting a 10% price premium in wool value. AFD received a substantially higher weight of 48, corresponding to a 40% price premium, reflecting its critical importance in the wool quality evaluation and the breeding program’s emphasis on this trait. To better align with current breeding objectives, the economic weights for SS and WWT were subsequently modified from their initial values (12 and 15.1, respectively) to 2 and 25 using the desired gains approach. This adjustment ensured that the expected genetic gains would be more consistent with practical breeding requirements.

### 3.2. Impacts of Index Trait Components and Their Sources of Information on the Index Selection Accuracy

The standard deviations ( $\sigma_{i}$ ) and selection accuracies ( $R_{IH}$ ) of the indices of three (A, B, and C) different component traits using the animal’s own information and/or information from its relatives per selection round assuming that the selection intensity was one are presented in Table 6.

The index with Group C traits expressed the best standard deviation and the highest accuracy while the source of information was the same since the Group C traits were identical to the breeding objective traits. For the index with Group A traits, compared with Group C, the only difference was that FDcv was applied to replace SS, which was a 2.5 to 3.8 CNY lower standard deviation and 3.6 to 5.4 percentile selection accuracy than Group C traits index using scenarios lacking offspring information. The additional use of offspring information in the Group A trait index decreased the differences in the standard deviation and selection accuracy to less than 1 CNY and approximately 1 percentile compared with the Group C index. Furthermore, since the index with B group traits was measured at assessment or shearing, the standard deviation and selection accuracy were, respectively, 10 CNY or more lower, and 15 to 16 percentiles lower than the index with A group traits. The result of comparison indicated that the index with B group traits showed the least selection efficiency, the index with C group traits the best, and the index with A group traits was in the middle but very close to the index with C group traits in terms of selection efficiency.

We observed the changes in the index standard deviation and the selection accuracy of two indices with A and B group traits (Table 6) with varying sources of information scenarios. The two parameters showed the lowest values when only the animal’s own information was used, and when the additional information from 10 half-sibs, sire, and dam in sequence were used, the index standard deviation increased within 3 CNY, and the selection accuracy increased in a range from 1 to 4 percentiles with the used of every additional information in sequence. On the basis of the combination of above-mentioned sources of information (④), the additional use of information from 16 progenies (⑤) resulted in increases in the index standard deviation of 10.6 CNY and 9.8 CNY, and increases in the selection accuracy of 15.1 and 14.1 percentiles, respectively, for the index with A and B group traits. Furthermore, the exclusion of the information on the sire and dam from scenario ⑤, that is, only the animal’s own information, 10 half-sibs, and 16 progenies were used (⑥), resulted only in reductions in the index standard deviation of 0.672 CNY and 0.633 CNY, and reductions in the selection accuracy of 1 and 0.9 percentile, respectively, for the index with A and B group traits. If we increased the number of progenies from 16 to 50 on the basis of the information source scenario ⑥, both the index standard deviation and the selection accuracy increased considerably. Since the available source of information in the current breeding context is limited, the number ⑥ scenario of source of information was preferentially recommended, and scenario ② was also recommended, while no information from progeny were available.

### 3.3. Impacts of the Flock Age Structure and Ewe Reproduction Rate on the Retention Rate and Selection Intensity

The numbers of sheep of different ages in the flock, and the corresponding average age, retention rate, selection intensity, and the ratio of selection intensity to average age (i/L) while the years of use ranged from 2 to 5 years for the sire and 3 to 6 years for dam in two cases when the ewe reproduction rate was, respectively, 85% and 95% are presented in Table 7. The results (Table 7) illustrated that with the reduction in the number of years of use of the sire and dam, the average age and the selection intensity of both the sire and dam decreased; however, the i/L ratio increased for the ram and decreased for the dam. According to Falconer’s statement [24], to pursue optimal genetic gains, the (im + if)/(Lm + Lf) ratio is what has to be maximized. This study estimated that the (im + if)/(Lm + Lf) were 0.44 and 0.43, respectively, when the dam was used for 5 and 6 years, assuming that all rams were used for 3 years. Furthermore, in this study, the annual genetic gains of breeding objectives in the four cases of the index and source of information scenarios (♂A②♀A②, ♂A②♀B②, ♂A⑥♀A②, and ♂A⑥♀B②) were compared between 5 and 6 years of use of the dam, assuming that the number of years of use of the ram was 3 years. The annual genetic gains of the breeding objective when the dam was used for 5 years were estimated to be 2.4% to 3.8% greater than the case when the dam was used for 6 years. So, the result suggested that the optimal years of use for the ram and ewe were, respectively, 3 and 5 years. The result also indicated (Table 7) that a 10% improvement in the ewe reproduction rate accompanied a reduced parental retention rate and increased selection intensity and i/L ratio.

### 3.4. Comparison of Selection Responses to Different Selection Indices

The weights of different index traits and different sources of information, the selection response of all traits involved, and the breeding objective per selection round when the years of use were 3 and 5 years, respectively, for the ram and ewe are presented in Table 8A, and Table 8B, respectively, for the index with A group traits and the index with B group traits. A comparison of selection responses between two indices with different group (A and B) traits was conducted in the case where their source of information was the same. The results of the comparison indicated that the selection response of the breeding objective of the index with A group traits was 30% greater than that of the index with B group traits when no progeny information was used, and the figure shrunk to 22% to 25% when the information of progeny was used. In comparison with the index with B group traits, index A increased the selection response of AFD and yield over 100% and 200%, respectively. The increase in the selection response for the traits CFW and SS of index A over index B, respectively, ranged from 20% to 42% and 8% to 15.6%. This prioity of the index with A group traits decreased with more sources of information used, particularly when the information from progeny were used, and the number of progenies’ sourcing information increased. On the other hand, for the body weight traits, the index with G group traits expressed more of a selection response for YWT and WWT than the index with A group traits. The selection responses of YSL, GFW and VFC were close to each other between the A and B indices. According to the priority the index of A group traits over the index with B group traits for the selection responses of the breeding objective and breeding objective traits, this study preferentially recommended index senario A② and secondly A⑥ for sire selection. For ewe selection, it preferentially recommended A② and secondly B②. For the selection of the ram and ewe, besides the animal’s own record, it is relatively easy to obtain 10 half-sibs’ information.

The annual genetic gains of the traits involved and the breeding objective under four combinations of recommended parental selection indices together with their source of information scenarios are presented in Table 9. The selection emphasis of breeding objective traits and the contribution rates from the sire and dam to the annual genetic gain of breeding objective are also presented in Table 9. The index selection scenario ♂A⑥♀A② can obtain the highest annual genetic gain for the traits AFD, CFW, FDcv, yield, and SS at, respectively, −0.223 μm, 0.062 kg, −0.187 percentile, 0.181 percentile, and 2.118 N/ktex. The annual genetic gains between the ♂A②♀A② and ♂A⑥♀B② index selection scenarios were very close to each other for AFD, CFW, FDcv, yield, and SS (Table 9). However, for the body weight traits YWT and WWT, the parental index selection scenarios ♂A⑥♀A② and ♂A⑥♀B②, in which information from progenies were included in the sire selection index, expressed better annual genetic gains than the scenarios ♂A②♀A② and ♂A②♀B②, in which no progeny information was used in the sire index selection. Given that the sire index selection scenarios were the same, the parental index selection scenario in which the dam used the index with B group traits was better than the scenario in which the dam used the index with A group traits for the two body weight traits. If the preferentially recommended parental index selection scenario ♂A②♀A② is adopted as the routine selection program, the accumulated 10-year genetic gain of breeding objective traits will be, respectively, −1.98 μm, 0.57 kg, 4.73 kg, 0.38 kg, 0.61 cm, and 20 N/ktex for AFD, CFW, YWT, WWT, YSL, and SS.

The selection emphasis of breeding objective traits presented in four parental index selection scenarios ranged from 40%~50% for AFD, which was the highest. The selection emphasis for SS was next to that for AFD, and then CFW, WWT, YSL, and YWT was the least. The contribution rates from ram to the annual breeding objective gains ranged from 74% to 82%, while those from dam ranged from 18% to 26%.

## 4. Discussion

### 4.1. Definition of the Breeding Objective

To pursue an overall goal of genetic improvement, defining a clear breeding objective and determining the relative value of trait improvement is the most important first step [25]. The first rule in the development of a breeding objective is that different breeding objectives are required for different breeding, production, and marketing systems in the commercial sector [26]. Average fiber diameter, clean fleece weight, and body weight, respectively, as the most important representative traits of wool quality, wool production, and growth, are included in the breeding objective of the superfine strain of Alpine Merino sheep, which is consistent with other Merino breeding practices [6,8,12]. In the breeding focusing on an improved AFD, more emphasis should be placed on simultaneously selecting for improved wool staple strength. Wool staple strength is the second most important wool quality trait next to AFD [27,28]. The negative genetic trend in the previous breeding stage necessitates incorporating the wool staple length to the breeding objective traits in the new breeding program. Both the weaning weight and yearling weight were incorporated in the breeding objective traits. Surplus weaners sold after weaning are a major income source of Alpine Merino sheep enterprises, while the weaning weight is the determinant of the price of weaners for sale. The yearling weight had positive genetic correlations with the fleece weight [19,29] and ewe reproduction rate [29], and a high yearling weight implicates a high body weight at culling, which was closely related to the income. In the Australian Merino breeding system, the wool fiber diameter and wool staple strength are included in objectives using a price premium [6,8], defined as the percent increase in price resulting from a unit change in the breeding objective trait. This price premium is constrained to range between 3% and 20% by the unfavorable genetic correlation between the fleece weight and fiber diameter [29] that allows the simultaneous improvement of both traits. Safari et al. concluded that the genetic correlations for AFD with CFW (0.28) and AFD with SS (−0.37) were unfavorable. However, in Alpine Merino sheep, the genetic correlation for AFD and CFW was −0.11 (unsignificant) and for AFD with SS was −0.74, which were all favorable, hence providing the theoretical possibility for the simultaneous improvement of the three traits [19]. In order to obtain a desirable genetic improvement of specialized traits in the superfine strain of Alpine Merino sheep, the price premium of AFD as a breeding objective was raised to 40% in this study, and the price premiums for SS and YSL were initially defined as 10%. The relevant economic weight for SS in breeding objective was 12, and the corresponding selection response was far from the practical context while using a 10% price premium for SS. So, the economic weight for SS was then modified to 2% using a desired gain approach [3,22,23] that makes the genetic gain of SS reach a more practical and desired range. The estimate in the previous study [19] for the genetic correlation between FDcv with SS was −0.91, which was consistent with the pooled mean of −0.68 in Safari’s review. Further research results illustrated that the indirect selection response for SS of selecting FDcv was almost equivalent to the direct response for SS [21]. Since there are not yet genetic parameters for reproduction traits available in Alpine Merino sheep, reproduction performances have not yet incorporated in breeding objectives of Alpine Merino sheep. It was suggested that indirect selection of reproduction rates is possible through body weight [6], which has a moderate positive genetic correlation with reproduction (0.33 [21]), and many ram breeders in effect rely on body weight as a proxy trait for reproduction [6]. The breeding objectives in this study also included body weights, which will ensure the genetic improvement of the reproduction of Alpine Merino sheep.

The contribution rates of different breeding objective traits to the overall breeding objective were seen as the emphasis rate on the specific breeding objective trait in the selection index. In the index FP+ currently used in Australia, which is aiming at improving wool fiber quality, the AFD contributes 46% to the overall economic income in the production system [30]. The figure is very close to the contribution rate (49.24%) of AFD to the breeding objective in superfine strain index in Alpine Merino sheep. However, the emphasis rate of CFW was 13.44% when both parents used the A② index scenario in this study, which was much lower than the same figure in the FP+ index in Australia. The emphasis rate on SS (20.77%) in this study was much higher than that (1%) in FP+. Other differences from FP+ were that we also put moderate emphases on yearling body weight (6.99%), weaning weight (4.87%), and yearling wool staple length (3.8%). The differences between our index and the FP+ index in Australia were mainly attributed to the breeding goal of superfine strain of Alpine merino sheep and to due to their differences in the genetic parameters of objective traits. According to the results of the measurement [21], there are practical requirement for improving the wool staple strength, wool staple length, and body weight of Alpine Merino sheep.

### 4.2. Improving Annual Genetic Gains by Adding Sources of Information for Selection

Optimal genetic gains can be obtained through selection by depending on the animal’s own information for those traits with high heritability in the index selection program. For the traits with low heritability, optimal genetic gains can also be obtained by adding information from their relatives, especially from their progenies. For the breeding objectives in Alpine Merino sheep in this study, except for staple strength, which has a high heritability, other traits are moderately heritable, such as AFD and CFW. In the selection context in Alpine Merino sheep, the most achievable practice is that the selection decisions are made at animals’ yearling age, depending on the trait information from the animal itself and its half-sibs, which may be much easier and allow measurement records to be obtained earlier. If the measurement of progeny’s performance was possible, it suggested that records from more progenies should be used for the breeding ram selection in order to improve the selection accuracy.

### 4.3. The Importance of Breeding Objective Trait Measurements to the Selection

The priority of the index with C group traits over A and B group traits in terms of selection accuracy and the genetic gain rate demonstrated the importance of the direct measurement of breeding objective traits to selection in this study. Nevertheless, the advantage of multi-trait index selection is that we can genetically improve those objective traits that are not easy to measure through index traits that may be easy to measure and genetically correlated with objective traits. Selection with the A group trait index, which included almost all objective traits, except using FDcv (genetic correlation with SS were −0.91 [21]) as a substitute for SS, only showed a minor difference from the selection with C group trait index, in which the index traits were the same as the objective traits, in terms of selection accuracy and genetic gain rate. Accordingly, the index with A group traits was preferentially recommended in this study. The index with B group traits, in which except for the traits WWT, YWT, and YSL, GFW and VFC were designed as substitutes, respectively, for CFW and AFD, attempting only use assessment and shearing records in the index, showed a much lower selection accuracy and genetic gain rate than the index with A group traits. The result demonstrated the importance of objective measurements to the genetic improvement of the breeding objectives. However, the index with B group traits also showed a desired genetic gain direction for the breeding objectives, and it provided an alternative option for the improvement of objective traits in the case where objective measurements for AFD and CFW are not available.

### 4.4. Improve Genetic Gains by Optimizing the Utilization Years of Rams and Ewes

Decreasing the years of utilization of stock animals (e.g., decreasing the culling age) can shorten the generation interval of breeding, which is used as one of the optional measures to improve genetic gain rate. But this measure may decrease the selection intensity as well. As illustrated in Table 1, for every one-unit decrease in utilization years of the parental animal, both the parental average age and the selection intensity decreased, but their decreases were not in the same proportions. If the magnitude of the decrease in the parental average age was larger than that of the selection intensity, then i/L will increase and hence increase the genetic gain rate. Conversely, the genetic gain rate will decrease. In the breeding context of Alpine Merino sheep, with every one-unit reduction in average utilization years, the ratio i/L of the sire increased, while the ratio i/L of the dam decreased. The reason for the decreased ratio i/L for the dam is that with the reduction in utilization years, the number of ewes retained for replacement increased, hence increasing the retention rate; as a result, the magnitude of the reduction in selection intensity became bigger than the magnitude of the reduction in the average parental age. So, precautions should be taken while attempting to improve the genetic gain rate by shortening the culling age of dams. The overall genetic gain depends on the selection intensity and average ages of both the sire and dam. The optimal utilization years of the sire and dam can be identified by the value of the ratio (im + if)/(Lm + Lf) [30] in the case where both the breeding ram and ewe use the same selection index. However, when the selection indices of the breeding ram and ewe are not the same, the optimal utilization years of sire and dam can be chosen through a comparison of pooled genetic gains. Meanwhile, the practical breeding management context should be considered when choosing the so-called optimal utilization year. For example, even if the i/L of the ram is the highest when the utilization year is two and hence the breeding ram flock is only composed of two-year-old and three-year-old rams, under the current sheep artificial insemination technology, it cannot satisfy the semen collection requirement for the insemination of the whole ewe flock. So, the optimal utilization years of the breeding rams and ewes chosen in this study were, respectively, 3 and 5 years, and were consistent with others [3,5].

### 4.5. Importance of More Emphasis on the Selection of Rams and Improvement of the Ewe Reproduction Rate for the Overall Genetic Gain

The contribution rates from both the sire and dam to the overall breeding objectives are solely depend on their respective selection intensity when the parents apply the same selection index with the same information source. In this study, when both parents applied the index with A group traits while using the information from the animal itself and its 10 half-sibs, the contribution rate of sire and dam were, respectively, 74% and 26%, of which the ratios were identical to the selection intensities of the sire (2.218) and dam (0.782). The result verified the importance of ram as an engine for the sheep industry, and so more emphasis should be placed on the ram selection and technical measures should be taken to increase the utilization rate of breeding rams to increase selection intensity of the sire and hence the genetic gain rate.

The additional use of progeny information could considerably improve the index selection accuracy and genetic gain rate of the ram in this study. Nevertheless, if the final selection decision for the breeding ram is made after the result of progeny test is available, then the generation interval will be surely prolonged [31]. So, as measures to the improve genetic gain rate, sire progeny tests and attempts to reduce sire utilization years can hardly be practiced simultaneously. As the recommended utilization year was three for sires in this study, a possible solution to this challenge is that the maiden rams (2 years old) can be intensively trained for semen collection prior to artificial insemination, so that their semen can be easily collected during AI, and not only can their semen satisfy the designed ewe flock insemination requirement but also enough progenies will be available for providing index selection information. In the third year of ram utilization, index values with additional progeny information can be used for ram selection decisions. Improvements in the ewe reproduction rate implied increased numbers of candidate animals for selection, a reduced parental retention rate, and increased selection intensity. The current study demonstrated the case in the breeding context of Alpine Merino sheep.

## 5. Conclusions

This study identified AFD, CFW, YSL, SS, YWT, and WWT as breeding objective traits in accordance with the breeding requirements of the superfine strain of Alpine Merino sheep, which were recommended as the optimal index component traits and the information from the candidate itself and 10 of its half-sibs were recommended as the most feasible sources of information for the selection of superfine strain of Alpine Merino sheep. It provided alternative options for the selection of the superfine Merino strain in varying breeding contexts. Suggestions were made to improve potential genetic gains by increasing the ewe reproduction rate, paying more attention to ram selection, and using the ram’s progeny information in the index selection.

## Figures and Tables

**Table 1:** Heritability and variability of traits and phenotypic and genetic correlations among the traits.

Trait	Unit	$\sigma_{p}$	$h^{2}$	$\sigma_{g}$		Genetic Correlations and Phenotypic Correlations									
						AFD	CFW	YWT	VFC	GFW	FDcv	WWT	YSL	YLD	SS
AFD	μm	1.48	0.204		0.67		0.08	0.18	−0.13	0.13	−0.05	0.07	0.08	−0.07	0.00
CFW	kg	0.43	0.201		0.19	−0.11		0.24	−0.05	0.77	0.02	0.27	0.37	0.54	0.25
YWT	kg	4.12	0.285		2.20	0.11	0.09		−0.07	0.36	−0.05	0.58	0.13	−0.03	0.24
VFC	counts	0.72	0.133		0.26	−0.23	−0.13	−0.22		−0.09	−0.08	−0.02	−0.19	−0.08	0.09
GFW	kg	0.67	0.186		0.29	0.01	0.74	0.21	−0.14		0.05	0.31	0.21	−0.05	0.08
FDcv	%	2.86	0.163		1.16	−0.08	0.24	0.09	−0.11	0.14		0.05	−0.01	−0.06	−0.42
WWT	kg	3.76	0.183		1.61	−0.04	0.13	0.63	−0.03	0.26	−0.04		0.11	−0.02	0.07
YSL	cm	1.00	0.189		0.44	−0.04	0.53	0.09	−0.04	0.38	0.11	0.08		0.23	0.18
YLD	%	5.49	0.352		3.26	−0.10	0.55	−0.12	−0.07	0.00	−0.10	−0.09	0.44		0.27
SS	N/ktext	13.62	0.415		8.77	−0.74	0.96	0.69	0.94	−0.05	−0.91	−0.36	0.44	0.33	

Notes: Values below the diagonal are genetic correlations. Values above the diagonal are phenotypic correlations.

**Table 2:** Simplified animal production model.

Primary Parameters	Number of Ewes (Heads)	Production per Ewe (kg)	Gross Production (kg)	Price (CNY/kg)	Income (CNY)	Cost (CNY)	Net Income (CNY)
Greasy fleece production	550	4.01	2205.50	30.00			
Clean fleece sold	550	2.48	1364.00	48.50	66,154.00		
Weaners sold	348	27.11	9420.73	24.00	22,6097.4		
Culled ewes sold	100	43.92	4392.00	15.94	70,008.48		
Net income per flock					36,2259.9	244,188.0	118,071.9
Net income per ewe					658.65	443.98	214.70

Notes: The total income was calculated from the sale of clean fleece weight, surplus weaners, and culled ewes, where the fleece yield was 61.85% and the reproduction rate of ewes was 85%. The income from weaners and culled ewes was calculated according to their live weight. The total cost included the expense of supplementary feeding, labor cost, and shared expenses of breeding ram feeding and management.

**Table 3:** Designed indices with their respective component traits.

Indices	YWT	WWT	YSL	AFD	CFW	VFC	GFW	FDcv	SS
A	√	√	√	√	√			√	
B	√	√	√			√	√		
C	√	√	√	√	√				√

Notes: “√” denotes the group of index traits in the row that includes the trait in the column.

**Table 4:** Different scenarios of sources of information of index traits with their relevant numbers.

Number Information Source	Own	10 Half-Sibs	Sire	Dam	16 Offsprings	50 Offsprings
①	√					
②	√	√				
③	√	√	√			
④	√	√	√	√		
⑤	√	√	√	√	√	
⑥	√	√			√	
⑦	√	√				√

Notes: “√” denotes the scenario of the source of information in the row that includes the source in the column.

**Table 5:** Marginal benefits of breeding objective traits and modified economic weights.

Objective Traits	Price Premium	Marginal Benefit	Economic Weights for the Breeding Objective
yearling weight	**/**	2.85	2.85
weaning weight	/	15.10	25.00
clean fleece weight	/	48.50	48.50
average fiber diameter	40%	48.00	48.00
staple strength	10%	12.00	2.00
staple length	10%	12.00	12.00

**Table 6:** Comparisons of standard deviations and selection accuracies among different indices.

	Index Traits	A		B		C		A-C		A-B	
Sources of Information		$\sigma_{i}$	$R_{IH}$	$\sigma_{i}$	$R_{IH}$	$\sigma_{i}$	$R_{IH}$	$\sigma_{i}$	$R_{IH}$	$\sigma_{i}$	$R_{IH}$
①		41.575	0.593	31.124	0.444	45.335	0.646	−3.760	−0.054	10.450	0.149
②		44.261	0.631	33.783	0.481	47.391	0.675	−3.130	−0.045	10.478	0.149
③		44.919	0.640	34.444	0.491	47.928	0.683	−3.009	−0.043	10.475	0.149
④		46.864	0.668	36.168	0.515	49.379	0.704	−2.514	−0.036	10.697	0.152
⑤		57.440	0.819	46.004	0.656	58.185	0.829	−0.745	−0.011	11.437	0.163
⑥		56.768	0.809	45.371	0.647	57.583	0.821	−0.815	−0.012	11.398	0.162
⑦		63.355	0.903	51.661	0.736	63.616	0.907	−0.261	−0.004	11.694	0.167

**Table 7:** Different flock age structures and corresponding retention rates and selection intensities.

Parents	Years of Use	Flock Size	Retained Number	Average Age (L)	Number of Animals of Different Ages						Reproduction Rate at 85%			Reproduction Rate at 95%		
					2	3	4	5	6	7	Retention Rate (%)	Selection Intensity (i)	i/L	Retention Rate (%)	Selection Intensity (i)	i/L
sire	2	120	61	2.49	61	59	0	0	0	0	5.22%	2.058	0.827	4.67%	2.083	0.837
	3	120	42	2.97	42	40	38	0	0	0	3.56%	2.218	0.747	3.19%	2.238	0.754
	4	120	32	3.45	32	31	29	28	0	0	2.72%	2.322	0.673	2.43%	2.358	0.683
	5	120	26	3.92	26	25	24	23	22	0	2.22%	2.384	0.608	1.99%	2.402	0.613
dam	3	550	191	2.97	191	183	176	0	0	0	81.71%	0.329	0.111	73.18%	0.450	0.152
	4	550	146	3.45	146	140	135	129	0	0	62.50%	0.606	0.176	55.98%	0.705	0.204
	5	550	119	3.92	119	114	110	105	101	0	50.99%	0.782	0.199	45.67%	0.868	0.221
	6	550	101	4.38	101	97	93	90	86	83	43.34%	0.907	0.207	38.81%	0.988	0.226

**Table 8:** (A) Index weights and responses to selection using the index of group A traits with varying sources of information. (B) Index weights and responses to selection using the index of group B traits with varying sources of information.

(A)													
Indices		A①			A②				A⑥				
		Weights (b)	Selection Response (R)		Weights (b)		Selection Response (R)		Weights (b)			Selection Response (R)	
Trait	Unit	Own	Trait	Objective	Own	10 Half-Sibs	Trait	Objective	Own	10 Half-Sibs	16 Offsprings	Trait	Objective
AFD	μm	−17.88	−0.440	21.141	−16.52	−15.53	−0.454	21.792	−8.70	−8.48	−46.32	−0.534	25.621
CFW	kg	23.99	0.129	6.278	22.30	12.18	0.131	6.344	10.99	6.87	44.67	0.146	7.069
YWT	kg	5.09	1.038	2.957	4.67	2.72	1.085	3.093	2.17	1.13	9.11	1.327	3.782
VFC	counts	-	0.147	-	-	-	0.148	-	-	-	-	0.161	-
GFW	kg	-	0.035	-	-	-	0.039	-	-	-	-	0.054	-
CV/FDcv	%	−1.82	−0.401	-	−1.68	−1.48	−0.403	-	−0.88	−0.80	−4.50	−0.438	-
WWT	kg	0.96	0.015	0.378	0.94	5.34	0.086	2.155	1.19	3.75	13.15	0.337	8.421
YSL	cm	4.91	0.137	1.642	4.55	5.01	0.140	1.683	2.50	2.82	14.37	0.163	1.957
YLD	%	-	0.385	-	-	-	0.386	-	-	-	-	0.425	-
SS	N/ktext	-	4.589	9.179	-	-	4.597	9.193	-	-	-	4.959	9.918
ϭ_I_				41.575				44.261					56.768
**(B)**													
**Indices**		**B** **①**			**B** **②**				**B** **⑥**				
		**Weights (b)**	**Selection Response (R)**		**Weights (b)**		**Selection Response (R)**		**Weights (b)**			**Selection Response (R)**	
**Trait**	**Unit**	**Own**	**Trait**	**Objective**	**Own**	**10 Half-Sibs**	**Trait**	**Objective**	**Own**	**10 Half-Sibs**	**16 Off-springs**	**Trait**	**Objective**
AFD	μm	-	−0.217	10.404	-	-	−0.222	10.667	-	-	-	−0.260	12.456
CFW	kg	-	0.091	4.405	-	-	0.095	4.606	-	-	-	0.117	5.655
YWT	kg	5.50	1.174	3.347	5.03	3.92	1.241	3.538	2.45	1.85	12.04	1.564	4.456
VFC	counts	19.79	0.140	-	18.33	20.53	0.144	-	10.23	12.23	59.96	0.170	-
GFW	kg	−2.74	0.020	-	−2.40	2.54	0.027	-	−0.33	2.71	5.16	0.052	-
CV/FDcv	%	-	−0.315	-	-	-	−0.318	-	-	-	-	−0.358	-
WWT	kg	0.50	0.136	3.406	0.54	3.60	0.208	5.194	0.85	2.73	9.06	0.461	11.513
YSL	cm	10.99	0.135	1.625	10.14	11.16	0.144	1.733	5.51	6.21	32.02	0.186	2.228
YLD	%	-	0.123	-	-	-	0.125	-	-	-	-	0.139	-
SS	N/ktext	-	3.969	7.938	-	-	4.022	8.045	-	-	-	4.532	9.063
ϭ_I_					31.124				33.783				45.371

Notes: The selection responses were calculated by adopting the scenario that the ram and ewe were, respectively, used for 3 and 5 years. 2. The information used for the calculation of the index was the phenotypic deviation from the mean or subgroup mean deviation of the phenotype from the flock mean.

**Table 9:** Annual genetic gains and selection emphases when parents adopted different index component traits with varying information sources.

Indices Used to Select Parents		♂A②♀A②			♂A②♀B②			♂A⑥♀A②			♂A⑥♀B②		
Trait	Unit	Trait Gain	H Gain (CNY)	Selection Emphasis (%)	Trait Gain	H Gain (CNY)	Selection Emphasis (%)	Trait Gain	H Gain (CNY)	Selection Emphasis (%)	Trait Gain	H Gain (CNY)	Selection Emphasis (%)
AFD	μm	−0.198	9.489	49.24%	−0.171	8.226	45.49%	−0.223	10.721	46.02%	−0.197	9.459	42.78%
CFW	kg	0.057	2.762	14.33%	0.053	2.565	14.18%	0.062	2.996	12.86%	0.058	2.798	12.66%
YWT	kg	0.473	1.347	6.99%	0.490	1.397	7.73%	0.550	1.569	6.73%	0.568	1.619	7.32%
VFC	counts	0.064	-	-	0.064	-	-	0.069	-	-	0.068	-	-
GFW	kg	0.017	-	-	0.016	-	-	0.022	-	-	0.020	-	-
CV/FDcv	%	−0.175	-	-	−0.166	-	-	−0.187	-	-	−0.177	-	-
WWT	kg	0.038	0.938	4.87%	0.051	1.283	7.10%	0.118	2.955	12.69%	0.132	3.300	14.93%
YSL	cm	0.061	0.733	3.80%	0.062	0.739	4.08%	0.068	0.821	3.52%	0.069	0.827	3.74%
YLD	%	0.168	-	-	0.139	-	-	0.181	-	-	0.151	-	-
SS	N/ktext	2.001	4.003	20.77%	1.936	3.873	21.42%	2.118	4.236	18.18%	2.053	4.106	18.57%
H gain/year	CNY		19.272	100.00%		18.083	100.00%		23.298	100.00%		22.109	100.00%
gain from sire	CNY		14.248	73.93%		14.248	78.80%		18.2747	78.44%		18.2747	82.66%
gain from dam	CNY		5.024	26.07%		3.834	21.20%		5.024	21.56%		3.834	17.34%

Notes: 1. In 4-character groups denote sire and dam selection indices and “♂” and “♀” refer to the sire and dam, respectively, following which “A” and “B’ represent the index component trait group adopted by either the sire or dam, the “②” denotes that the index component trait information was from the animal’s own information plus that of 10 half-sibs, and “⑥” denotes the information sources of ② plus an additional 16 offsprings’ performance. 2. The selection responses were calculated by adopting the scenario that ram and ewe were, respectively, used for 3 and 5 years.

## Data Availability

The data presented in this study are available upon request from the corresponding author due to privacy.

## Abbreviations

The following abbreviations are used in this manuscript:

AFD	Average fiber diameter
CFW	Clean fleece weight
YWT	Yearling body weight
VFC	Visual fineness counts
GFW	Greasy fleece weight
FDcv	Coefficient of variation of AFD
WWT	Weaning body weight
YSL	Yearling staple length
YLD	Yield
SS	Wool staple strength
$\sigma_{i}$	Standard deviation
$R_{IH}$	Selection accuracy
